# Supplementary material for: Cytolytic circumsporozoite-specific memory CD4+ T cell clones are expanded during Plasmodium falciparum infection
Source: Nat Commun. 2023 Nov 25;14:7726. doi: 10.1038/s41467-023-43376-y (PMC10673885; doi:10.1038/s41467-023-43376-y)
Supplement: Supplementary file 1 — Supplementary Information [file 41467_2023_43376_MOESM1_ESM.pdf]

Supplementary Information

Cytolytic circumsporozoite-specific memory $CD4^+$ T cell clones are expanded during *Plasmodium falciparum* infection

Raquel Furtado,[1#11] Mahinder Paul,[1#] Jinghang Zhang,[1] Joowhan Sung,[2] Paul Karell,[1] Ryung S. Kim,[3] Sophie Caillat-Zucman,[4] Li Liang,[5] Philip Felgner,[5] Andy Bauleni,[6] Syze Gama,[7] Andrea Buchwald,[8] Terrie Taylor,[7,9] Karl Seydel,[7,9] Miriam Laufer,[8] Fabien Delahaye,[10 12] Johanna P. Daily,[1,2]* Grégoire Lauvau[1]*

[1]Department of Microbiology and Immunology, [2]Department of Medicine, [3]Department of Epidemiology and Population Health, [10]Department of Genetics, Albert Einstein College of Medicine, Bronx, New York, USA 10461

[4]Université de Paris, AP-HP, Hôpital Saint-Louis, Laboratoire d'Immunologie et Histocompatiblité, INSERM UMR976, 75010 Paris, France

[5]Department of Physiology and Biophysics, School of Medicine, University of California, Irvine, California, USA 92697.

[6]Malaria Alert Centre, Kamuzu University of Health Sciences, Blantyre, Malawi

[7]Blantyre Malaria Project, Kamuzu University of Health Sciences, Blantyre, Malawi

[8]Center for Vaccine Development and Global Health, University of Maryland School of Medicine, Baltimore, Maryland, USA 21201

[9]Department of Osteopathic Medical Specialties, Michigan State University, East Lansing, Michigan, USA 48824

Supplementary Figures and Data

Supplementary Figure 1, related to Figure 1.

Supplementary Figure 2, related to Figure 2.

Supplementary Figure 3, related to Figure 3 and 4.

Supplementary Figure 4, related to Figure 5.

Supplementary Figure 5, related to Figure 6.

Supplementary Figure 6, related to Figure 7.

Supplementary Data 1: List of IgG reactive *Pf* antigens.

Supplementary Data 2: List of *Pf* -reactive antigens between patient's groups.

Supplementary Data 3: List of IgG-specific *Pf* antigens with greater reactivity in protected compared to susceptible children.

Supplementary Data 4: List of antibodies for CyTOF, spectral flow cytometry and AIM assay.

Supplementary Data 5: List of expressed genes in naive and memory $CD4^+$ T cell clusters defined by single cell transcriptomic.

A

Participant enrollment characteristics by age

Variable	1-5 yr (n=35)	6-12 yr (n=33)	13-50 yr (n=27)	*P* value (ANOVA)
Sex (% female)	40	61	70	<0.05†
Temperature (°C)	38.0 (36.7-39.1)	38.1 (36.25-38.8)	36.4 (35.9-36.6)	<0.0001
Parasitemia (x10^3/µl)	38.2 (15.9-79.9)	18.86 (1.4-62.8)	0.88 (0.5-6.5)	<0.0001
Hemoglobin (g/dL)	10.3 (9.3-11.7)	11.5 (10.8-12.2)	12.6 (10.9-1.0)	<0.0001

*Median and IQR
† Chi-squared test

B **C**

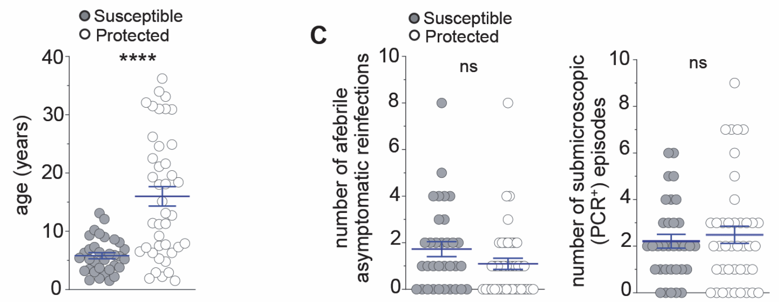

D

Participant enrollment characteristics by clinical immunity status and age

Variable	Susceptible, <13 yr (n=32)	Protected, <13 yr (n=20)	Protected, ≥13 yr (n=21)	*P* value (ANOVA)
Age, (yr)	5.2 (3.3-7.2)	6.9 (3.4-8.9)	24.6 (18.9-31.3)	NA
Sex (% female)	56	30	71	0.027†
Temperature (°C)	38.4 (36.4-39.2)	38.0 (36.2-39.1)	36.3 (36.0-36.7)	0.002
Parasitemia (x10^3/µl)	32 (4.2-68)	21 (2.2-66)	0.7 (0.4-4.1)	0.003
Hemoglobin (g/dL)	10.7 (9.9-12.1)	11.4 (10.8-12.2)	12.8 (11.6-14.1)	0.001

*Median and IQR
† Chi-squared test

E

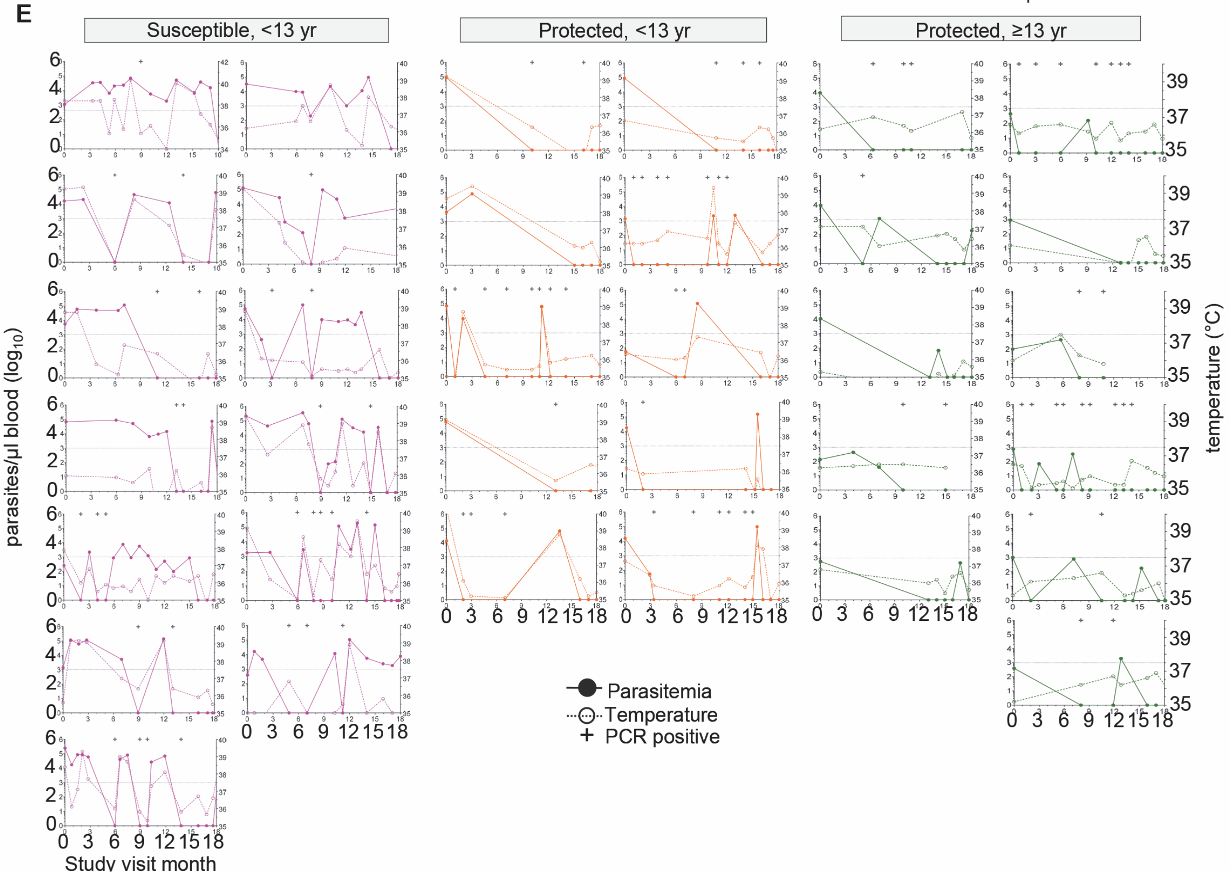

Supplementary Figure 1. Clinical characteristics of the mild malaria cohort in the 18-month longitudinal study: (A) Table with participant characteristics and clinical parameters at enrollment in the study, categorized by participants' age. Median and IQR values, and p-value determined by two-way ANOVA statistical analysis across participant's groups is indicated. **(B)** Average number of days between clinical reinfection episodes (>2,500 parasites/µl blood) and age (years) of each participant that completed the study within the protected (≤1 clinical reinfection, n=41) and susceptible groups (≥3 clinical reinfections, n=33). **(C)** Number of afebrile asymptomatic reinfections (<2,500 parasites/µl blood, temperature <37.5°C) and submicroscopic episodes (*Pf* PCR$^+$) two sided Student's t-test was conducted between indicated groups, *p<0.05, **p<0.01, ***p<0.001, ****p<0.0001. **(D)** Table participant enrollment characteristics by clinical immunity status and age. **(E)** Parasitemia (left axis), temperature (right axis) and *Pf* PCR status of a subset of susceptible, <13 yr, protected, <13 yr and protected, ≥13 yr at each clinical visit over the 18-month longitudinal study. Data are presented as means ± SEM (B,C).

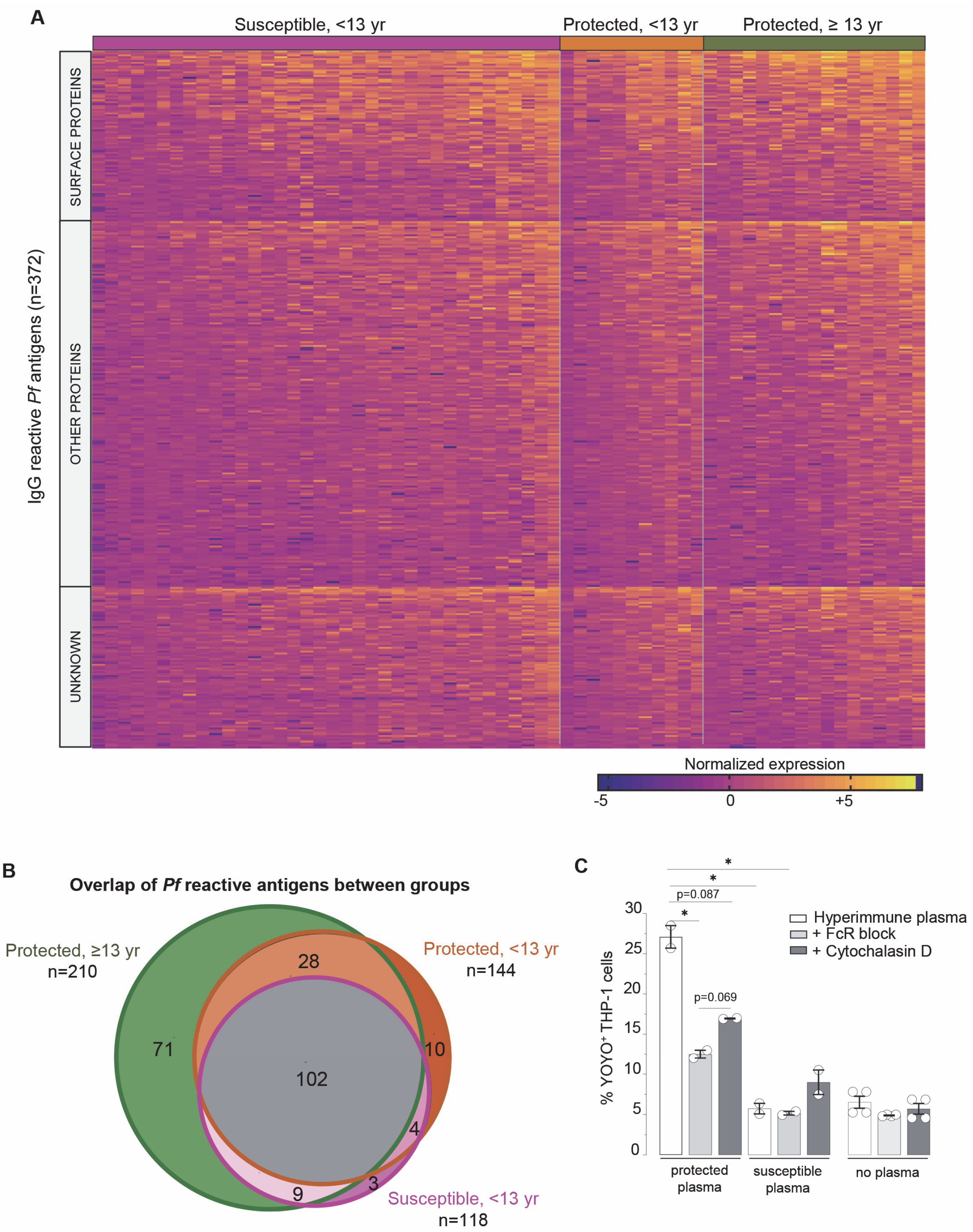

Supplementary Figure 2. *Pf* specific antibody responses in participants determined by *Pf* antigen array: (A) Heatmap of normalized intensity of IgG reactivity to 372 *Pf* antigens (rows)

in plasma from individual participants (columns) within susceptible, <13 yr, protected, <13 yr and protected, ≥ 13 yr groups during malaria illness. Surface and non-surface *Pf* antigens are indicated. **(B)** Venn diagram indicating overlapping *Pf* IgG reactivity to 372 *Pf* Ags by susceptible n=32, protected age-matched (n=20) and protected > 13 yr groups (n=21). Total *Pf* Ags recognized by each group is indicated next to the group label. **(C)** *Pf* merozoite opsonization assay with YOYO1^{+} *Pf* merozoites incubated with or without indicated plasma (1:9 dilution, duplicate wells) and co-cultured with THP1 cells in the presence or absence of FcR block or Cytochalasin D (n=2). Mean and SEM of the % YOYO1^{+} THP-1 cells are shown. Statistics is two sided Welch's unpaired t-test between indicated groups *$p<0.05$. Data are presented as means ± SEM (C).

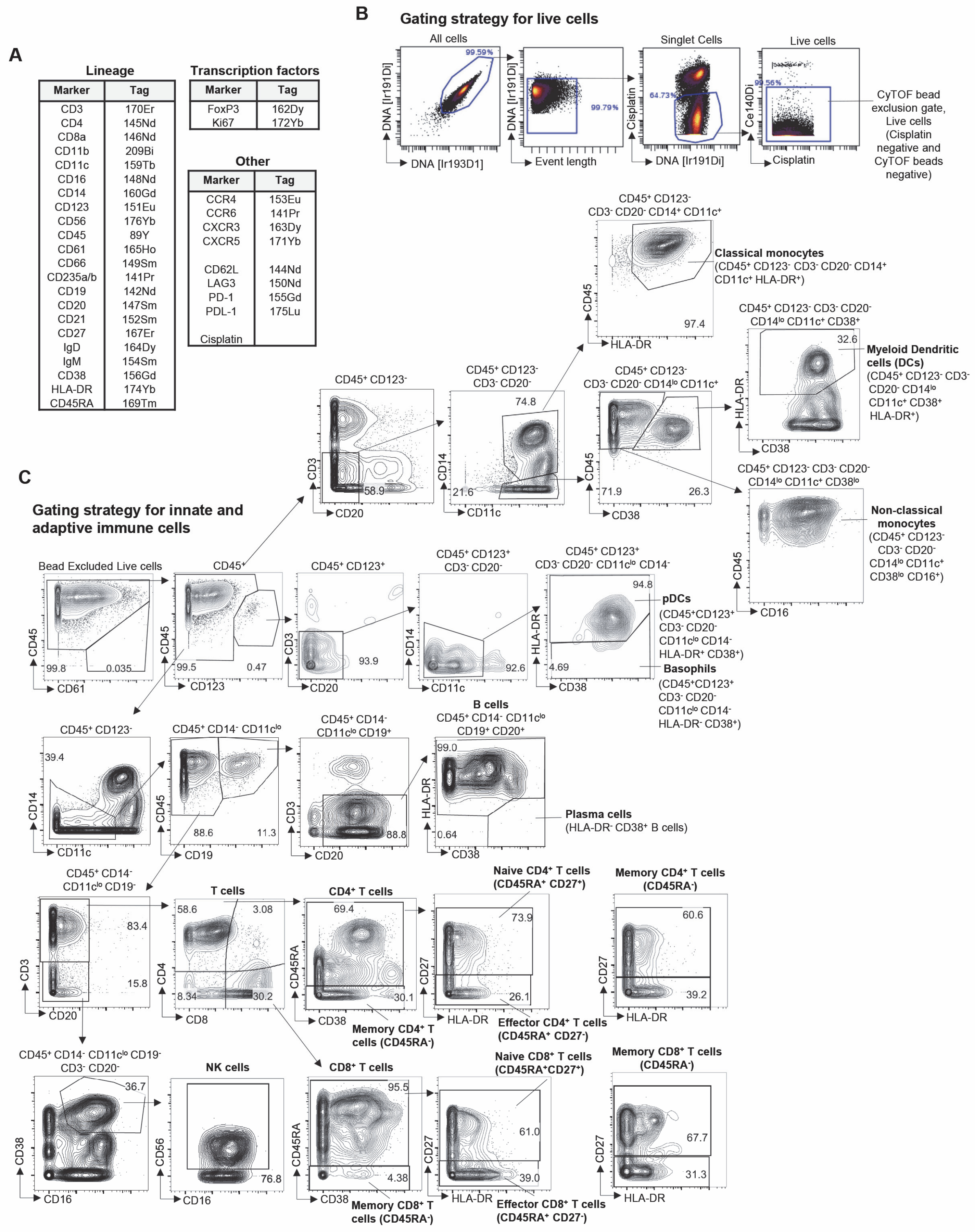

Supplementary Figure 3. CyTOF immunophenotyping panel and gating strategy of immune cell populations: (A) Participant PBMCs were immunophenotyped with a 33 marker

mass cytometry antibody panel with indicated heavy-metal tags. **(B)** Gating strategy for live cells among PBMCs immunophenotyped by CyTOF. A density plot that is a representative sample during malaria illness is shown. **(C)** Contour plots with gating strategy for innate and adaptive immune cell populations among PBMCs stained with the antibody panel in (A).

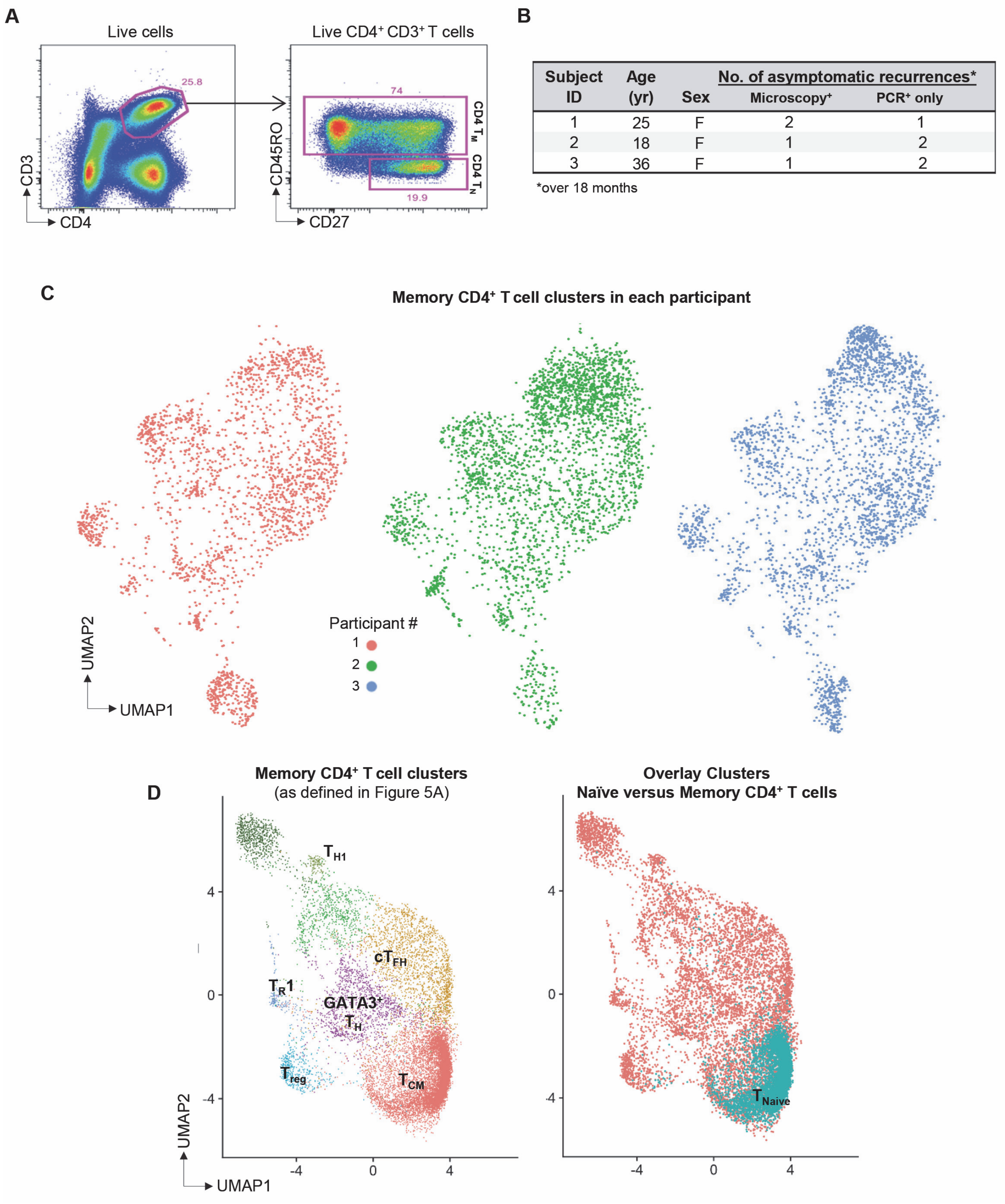

Supplementary Figure 4. Single-cell (sc) transcriptomic analysis of memory and naive CD4+ T cells from protected participants during malaria illness: (A) Dot plots show the gating strategy for sorting live (L/D aqua negative), memory (CD45RO+) and naïve (CD45RO-

CD27^{+}) CD4^{+} T cells on the BD Aria III for scRNA-seq analysis. **(B)** Table of the characteristics of the three protected participants from which PBMC were used for sc transcriptomic and TCR-seq. **(C)** UMAP visualization of scRNA-seq results on the memory CD4^{+} T cells from each of the 3 protected participants during malaria infection. **(D)** UMAP visualization of scRNA-seq results on isolated naïve versus memory CD4^{+} T cells from 3 protected participants during malaria infection. **(D)** UMAP visualizations of i) memory (CD45RO^{+}) clusters as defined in Figure 5A (left) and ii) overlay of naive and memory CD4^{+} T cell distributions from the 3 protected participants during malaria infection (right).

A

	TCR Vα	CDR3α	TCR Vβ	CDR3β	Proportion (%)
Participant 1	TRAV25	CAGRAAGNKLTF	TRBV6-3	CASIHQGQLEAFF	4.35
	-	-	TRBV6-3	CASIHQGQLEAFF	1.85
	TRAV12-3	CAMSAGYGGSQGNLIF	TRBV27	CASRAGHLYEQYF	1.25
	TRAV12-2	CAANPEGNQFYF	TRBV5-4	CASSFGGSSSYEQYF	1.18
	TRAV20	CAVHRGGGSQGNLIF			
	TRAV14DV4	CAMRDGGGAQKLVF	TRBV5-1	CASSTGTGLNSGANVLTF	1.11
	TRAV26-1	CIVRVTPSWGKLQF	TRBV7-2	CASSLASGGYNEQFF	0.98
	TRAV9-2	CARKNTGFQKLVF	TRBV7-8	CASSLDGTGSYEQYF	0.94
	TRAV29DV5	CAASGNAGNMLTF	TRBV7-9	CASSRTSGWGNEQFF	0.91
	TRAV12-3	CAMSAGYGGSQGNLIF			
	TRAV41	CAHPPYGSRLTF	TRBV27	CASRPGHYYEQYF	0.54
	TRAV1-2	CAVRDPTGGGNKLTF	TRBV29-1	CSVEVGSKEYF	0.34
Participant 2	TRAV9-2	CALSAKGAQKLVF	TRBV5-1	CASDLGHRDRAYEQYF	1.97
	-	-	TRBV5-1	CASDLGHRDRAYEQYF	1.01
	TRAV4	CLVGFMDTGRRALTF	TRBV12-5	CASGLGRTGTDTQYF	0.35
	TRAV2	CAVRVGGYNKLIF	TRBV19	CASSIDQGGGAVEQFF	0.32
	TRAV29DV5	CASMDTGRRALTF	TRBV6-5	CASSYSATGPLHF	0.29
	-	-	TRBV19	CASSIDQGGGAVEQFF	0.29
Participant 3	TRAV41	CAVSNTGNQFYF	TRBV5-5	CASSLRGLDYEQYF	2.39
	-	-	TRBV5-5	CASSLRGLDYEQYF	1.22
	TRAV13-1	CAASIRFQAGTALIF	TRBV3-1	CASSQGASGGETQYF	0.94
	-	-	TRBV3-1	CASSQGLGAGNTIYF	0.56
	TRAV1-2	CASYQNFVF	TRBV3-1	CASSQGLGAGNTIYF	0.5
	TRAV8-2	CVVSGISGNTPLVF	TRBV3-1	CASSQVAGANYGYTF	0.5
	TRAV1-2	CAVRDPSSGSARQLTF	TRBV5-1	CASSPVGAETQYF	0.33
	TRAV9-2	CALSDQEAGTALIF	TRBV2	CASRGSTQYQETQYF	0.33
	-	-	TRBV3-1	CASSQVAGANYGYTF	0.28

B

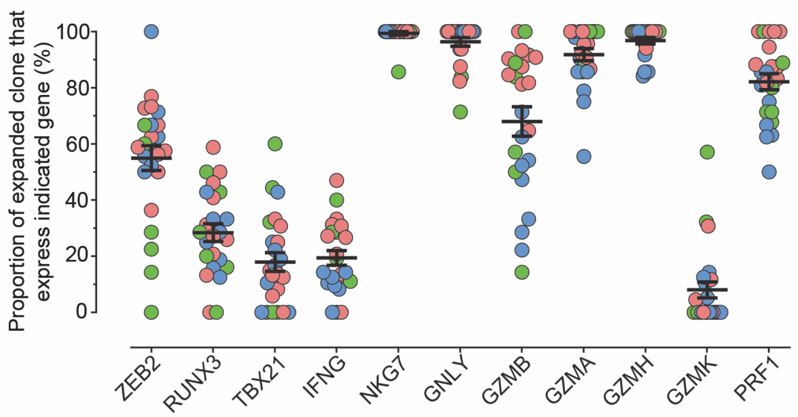

C

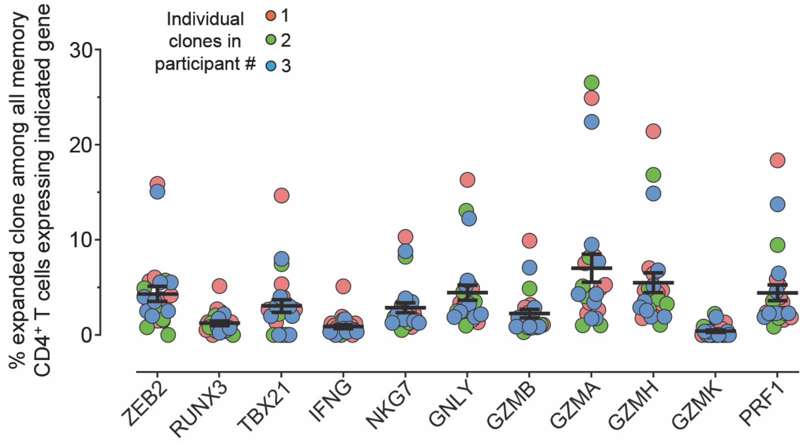

Supplementary Figure 5. Additional characterization of expanded memory CD4$^+$ T cell clones of the 3 protected participants: **(A)** Variable region sequences of the TCRα and TCRβ chains in expanded memory CD4$^+$ T cell clones and their frequencies in protected malaria participants (n=3), defined by single-cell TCR-seq. **(B)** Relative expression of indicated gene in each individual expanded memory CD4$^+$ T cell clone. **(C)** Frequency of expanded memory CD4$^+$ T cell clone among all memory CD4$^+$ T cells that express the indicated gene in the 3 protected participants.

A **Characteristics of study subjects for AIM assay**

Variable	Median and IQR n=15
Age, (yr)	5.2 (2.9-9)
Sex (% female)	33
Number symptomatic events	1 (0-3)
Number all recurrent events	6 (3-9)

#Symptomatic malaria ≥ 2500 parasites/ul and ≥ 37.5°C

B

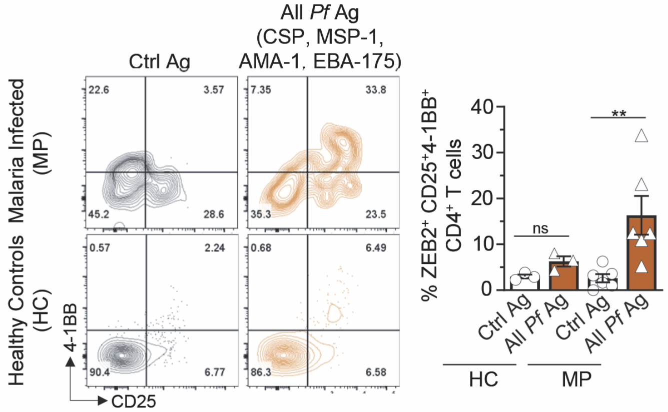

C

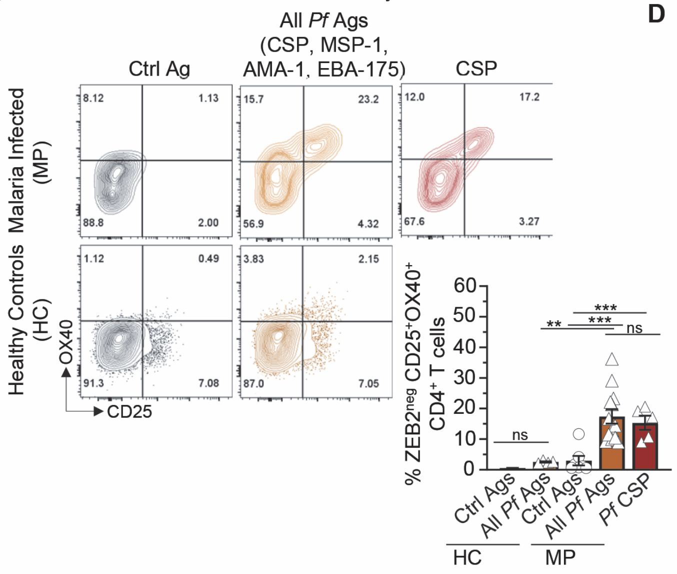

D

Lineage

Marker	Tag
CD3	AF532
CD4	Pacific Orange
CD14	Qdot-705
CD45	Qdot-800
CD19	BUV737
CD27	PerCP
CD45RO	BV570

Transcription factors

Marker	Tag
ZEB2	AF700

Other

Marker	Tag
CCR6	BV711
CX3CR1	PE Dazzle 594
CXCR3	BV650
CXCR5	Super Bright 436
CD25	PE-Cy5
CD39	APC Fire750
CD62L	BV480
CD127	BUV750
IgG	BUV395
Granzyme B	PE-Cy5.5
ICOS	AF488
LAG3	PE-Cy7
PD-1	BB515
OX40	BB700
TIGIT	BV605
Live/Dead	Zombie NIR

E **Characteristics of study subjects analyzed during repeated infection**

Variable	Not gaining protection (n=6)	Gaining protection (n=5)	*P* value
Age, (yr)	2.7 (1.6-4.4)	6.2 (4.6-7.6)	0.026
Sex (% female)	50	50	>0.999†
Number symptomatic events	4.5 (2.5-5.3)	1.0 (1.0-1.3)	0.015
Number all recurrent events	11.5 (8.5-13.5)	8 (4.5-11)	0.093

*Median and IQR
† Fisher's exact test
#Symptomatic malaria ≥ 2500 parasites/ul and ≥ 37.5°C

Supplementary Figure 6. AIM assay on ZEB2^{+} and ZEB2^{neg} memory CD4^{+} T cells in participants during illness: (**A**) Table summarizing the characteristics of the study participants whose PBMC were used for the AIM assay. (**B**) Representative FACS dot plots of CD25 and 4-1BB expression by ZEB2^{+} memory CD4^{+} T cells in i) Healthy controls (HC) stimulated with control antigen (ctrl Ag) (n=3) or all *Pf* Ag (Circumsporozoite Protein (CSP), Merozoite Surface Protein-1 (MSP-1), Apical Membrane Antigen 1 (AMA-1), Erythrocyte Binding Antigen 175 (EBA-175) (n=3) and in ii) Malaria Patients (MP) stimulated with ctrl Ag (n=6) or all *Pf* Ag (n=6). (**C**) Representative FACS dot plots of CD25 and OX40 expression by ZEB2^{neg} memory CD4^{+} T cells in i) HC stimulated with ctrl Ag (n=3) or all *Pf* Ag (n=4) and in ii) MP ctrl Ag (n=7), all *Pf* Ag (n=13) or *Pf* CSP (n=5). Bar graphs pool 3 independent experiments across distinct participants, each symbol is one participant. Two-sided Student's t-test was conducted between indicated groups ns- non-significant, *$p<0.05$, **$p<0.01$, ***$p<0.001$. (**D**) Spectral flow cytometry panel (Aurora, Cytek) used for phenotypic characterization of PBMCs in Figures 7 and 8. **(E)** Table summarizing the characteristics of the study participants who gain or do not gain clinical immunity during the study.
