# Supplementary material for: Cytolytic circumsporozoite-specific memory CD4+ T cell clones are expanded during Plasmodium falciparum infection
Source: Nat Commun. 2023 Nov 25;14:7726. doi: 10.1038/s41467-023-43376-y (PMC10673885; doi:10.1038/s41467-023-43376-y)
Supplement: Supplementary file 3 — Description of Additional Supplementary Files [file 41467_2023_43376_MOESM3_ESM.pdf]

Description of Additional Supplementary Files

File Name: Supplementary Data 1
Description: List of IgG reactive *Pf* antigens.

File Name: Supplementary Data 2
Description: List of *Pf* -reactive antigens between patient's groups.

File Name: Supplementary Data 3
Description: List of IgG-specific *Pf* antigens with greater reactivity in protected compared to susceptible children.

File Name: Supplementary Data 4
Description: List of antibodies for CyTOF, spectral flow cytometry and AIM assay.

File Name: Supplementary Data 5
Description: List of expressed genes in naive and memory $CD4^+$ T cell clusters defined by single cell transcriptomic.

File Name: Supplementary Code 1
Description: Detailed script used in R software for the single cell analysis.
