# Supplementary material for: Cytolytic circumsporozoite-specific memory CD4+ T cell clones are expanded during Plasmodium falciparum infection
Source: Nat Commun. 2023 Nov 25;14:7726. doi: 10.1038/s41467-023-43376-y (PMC10673885; doi:10.1038/s41467-023-43376-y)
Supplement: Supplementary file 10 — Reporting Summary [file 41467_2023_43376_MOESM10_ESM.pdf]

nature portfolio

Corresponding author(s): Gregoire Lauvau, Johanna Daily

Last updated by author(s): Oct 24, 2023

Reporting Summary

Nature Portfolio wishes to improve the reproducibility of the work that we publish. This form provides structure for consistency and transparency in reporting. For further information on Nature Portfolio policies, see our Editorial Policies and the Editorial Policy Checklist.

Statistics

For all statistical analyses, confirm that the following items are present in the figure legend, table legend, main text, or Methods section.

n/a	Confirmed	
☐	☒	The exact sample size (*n*) for each experimental group/condition, given as a discrete number and unit of measurement
☐	☒	A statement on whether measurements were taken from distinct samples or whether the same sample was measured repeatedly
☐	☒	The statistical test(s) used AND whether they are one- or two-sided<br>*Only common tests should be described solely by name; describe more complex techniques in the Methods section.*
☒	☐	A description of all covariates tested
☐	☒	A description of any assumptions or corrections, such as tests of normality and adjustment for multiple comparisons
☐	☒	A full description of the statistical parameters including central tendency (e.g. means) or other basic estimates (e.g. regression coefficient) AND variation (e.g. standard deviation) or associated estimates of uncertainty (e.g. confidence intervals)
☐	☒	For null hypothesis testing, the test statistic (e.g. *F*, *t*, *r*) with confidence intervals, effect sizes, degrees of freedom and *P* value noted<br>*Give P values as exact values whenever suitable.*
☒	☐	For Bayesian analysis, information on the choice of priors and Markov chain Monte Carlo settings
☐	☒	For hierarchical and complex designs, identification of the appropriate level for tests and full reporting of outcomes
☐	☒	Estimates of effect sizes (e.g. Cohen's *d*, Pearson's *r*), indicating how they were calculated

Our web collection on statistics for biologists contains articles on many of the points above.

Software and code

Policy information about availability of computer code

Data collection	Flow analysis was performed using FACSDIVA version 8.0.1 (BD Pharmingen). Flow sorting was performed using FACSDIVA versions 6.1.3 or 8.0.2 (BD Pharmingen). Sequencing data was collected on a Hi-seq Illumina platform.
Data analysis	-Statistical analysis was carried out using GraphPad Prism (v9).<br>-All flow cytometry data were analyzed using FlowJo software (TreeStar v9 or v10).<br>-Single cell RNA-seq analysis and differential expression analysis: Unique Molecular Index (UMI) Count Matrices for gene expression were generated using the CellRanger count (Feature Barcode) pipeline (v2.1.1). Reads were aligned on the GRCh38-3.0.0 transcriptome reference (10x Genomics). Chemistry was Single Cell 5' R2-only. Filtering for low quality cells according to the number of RNA, genes detected, and percentage of mitochondrial RNA was performed. Then, gene expression matrix was regress for cellular sequencing depth and mitochondrial percentage using linear modeling as implemented in Seurat ScaleData function.<br>Dataset were integrated within each condition using the Seurat (v3) Canonical Correlation Analysis (CCA) and graph-based integration tool on the 3,000 most expressed genes across datasets to correct for batch effect. The 30 first dimensions of the PCA of the batch effect corrected matrix was used to generate the Shared Nearest-neighbor (SNN) graph and the UMAP. Graph-based clustering using Louvain algorithm with a resolution parameter of 0.46 on the FindCluster function was used to cluster cells. Each cluster was annotated using cell type specific markers. Markers for each cluster were identified using FindAllMarkers function with default parameter. Genes were then ranked based on their expression fold change the difference of detection of this gene in the cluster versus all other clusters and the specificity for the cluster, and top cluster-specific genes were compared with published cell type-specific genes to annotate cluster. Up and down regulated genes for each cluster were identified using likelihood-ratio test for single-cell gene expression comparing cluster of interest with all other clusters. P-values for reported genes were <0.01.<br>-Single cell RNA-seq TCR analysis: Raw data of the TCR libraries were first processed with the CellRanger VDJ pipeline (ChemistrySingle Cell V(D)J). We used V(D)J Reference GRCh38-alts-ensembl and Cell Ranger Version2.1.1. The output TCR sequences in each cell were integrated

with their expression data using the shared cell barcodes within each sample. Downstream analyses were applied in R/ Seurat package (v3.2.2). We use clonotype frequency distribution to define clonally expanded cells. The threshold used based on the distribution tail was a clonotype frequency greater than 10. Differential expression analysis between expanded versus non expanded was performed using likelihood-ratio test for single-cell gene expression. P-values for reported genes were <0.01.
Full details are described in the Methods section of this paper.
-Pseudotime analysis: Differentiation trajectory analyses were conducted with monocle71 (https://www.bioconductor.org/packages/monocle/). Preprocessed Seurat object were imported using importCDS function from the monocle R package. Monocle's orderCells function was used to arrange cells along a pseudo-time axis to indicate their position in a differentiation continuum. Monocle generates for each cell a pseudotime value in respect to predefined cell of origins (roots). Here the TCM cells were used as roots. We specify the root of the trajectory programmatically, as recommended, by first grouping the cells according to which trajectory graph node they are nearest to. Then, calculating what fraction of the cells at each node come from the earliest time point followed by selecting the node that is most heavily occupied by early cells and returns that as the root.

For manuscripts utilizing custom algorithms or software that are central to the research but not yet described in published literature, software must be made available to editors and reviewers. We strongly encourage code deposition in a community repository (e.g. GitHub). See the Nature Portfolio guidelines for submitting code & software for further information.

Data

Policy information about availability of data

All manuscripts must include a data availability statement. This statement should provide the following information, where applicable:

- Accession codes, unique identifiers, or web links for publicly available datasets
- A description of any restrictions on data availability
- For clinical datasets or third party data, please ensure that the statement adheres to our policy

The accession number for the scRNA-seq and TCR-seq data reported in this paper is GEO:GSE182536. All data is available in the main text or the supplementary materials.

Research involving human participants, their data, or biological material

Policy information about studies with human participants or human data. See also policy information about sex, gender (identity/presentation), and sexual orientation and race, ethnicity and racism.

Reporting on sex and gender	Findings apply to both sexes. Sex was considered in the study design. Sex was based on self reporting, or in the case of children based on the guardian or parents reporting. Supplemental figure S1D reports clinical immunity status by sex. All data is de-identified.
Reporting on race, ethnicity, or other socially relevant groupings	This study was conducted in Malawi. All study participants are African.
Population characteristics	Basic demographics of the study populations are presented. (age, sex, number of malaria infections over 18 months, sickle cell status-based on genotype)
Recruitment	Patients were recruited to the study at one health care center in Malawi, under the auspices of the Malawi International Center of Excellence for Malaria Research; The study was explained to all potential study subjects, in the local language of Chichewa and consent was obtained for those who enrolled. We enrolled Malawian children, adolescents and adults, and over sampled younger participants to provide a sufficient number of subjects who displayed variation in clinical immunity. Patients were prospectively studied to identify those with clinical immunity and those without clinical immunity. Prospective studies have less sources of bias compared to retrospective studies, and we did not identify overt issues that may have biased the study and negatively mpacted the results.
Ethics oversight	Institutional Review Board approvals were obtained from the Albert Einstein College of Medicine, Michigan State University, the University of Maryland, and the University of Malawi College of Medicine Research and Ethics Committee.

Note that full information on the approval of the study protocol must also be provided in the manuscript.

Field-specific reporting

Please select the one below that is the best fit for your research. If you are not sure, read the appropriate sections before making your selection.

☒ Life sciences ☐ Behavioural & social sciences ☐ Ecological, evolutionary & environmental sciences

For a reference copy of the document with all sections, see nature.com/documents/nr-reporting-summary-flat.pdf

Life sciences study design

All studies must disclose on these points even when the disclosure is negative.

Sample size	To obtain sufficient number of study participants who display susceptibility and protection from clinical malaria, and to allow subanalysis by

Sample size | sex, we examined previously published studies of clinical malaria in residents of Malawi (a highly malaria endemic country). This provided the basis for our sample size calculations. We enrolled 120 study subjects that varied in age, and 97 completed the study. We were able to discriminate two large groups of subjects into a Susceptible and Protected group with high statistical significance with this sample size.

Data exclusions | (1) Subjects who did not complete the 18 month prospective study were excluded (n=27); (2) The sickle cell mutation protects against clinical malaria and is a confounder to our study of immune correlates of protection, thus we excluded the two study subjects who had sickle cell mutation.

Replication | Immunophenotyping was performed using CyTOF on independent clinical sample once. For key findings, we recapitulated the immunophenotyping using Cytek Aurora spectral flow cytometry on an additional aliquot of PBMCs from the study subjects. For our in vitro functional assay (AIM assay) we used PBMC samples from six study subjects. Experiments were recapitulated twice successfully with similar results.

Randomization | This was an observational clinical study; subjects were not randomized to any intervention. For our immune analysis, age is a known confounder that is associated with protection against clinical malaria; thus we conducted a sub-study to assess immune responses in aged matched (children < 13 yrs of age) that were categorized as susceptible or protected against clinical malaria.

Blinding | Investigators were blinded to the study group allocation (susceptible or protected) for all of the assays reported.

Reporting for specific materials, systems and methods

We require information from authors about some types of materials, experimental systems and methods used in many studies. Here, indicate whether each material, system or method listed is relevant to your study. If you are not sure if a list item applies to your research, read the appropriate section before selecting a response.

Materials & experimental systems

n/a	Involved in the study	
☐	☒	Antibodies
☐	☒	Eukaryotic cell lines
☒	☐	Palaeontology and archaeology
☒	☐	Animals and other organisms
☐	☒	Clinical data
☒	☐	Dual use research of concern
☒	☐	Plants

Methods

n/a	Involved in the study	
☒	☐	ChIP-seq
☐	☒	Flow cytometry
☒	☐	MRI-based neuroimaging

Antibodies

Antibodies used | All information is provided in the Supplementary Table 4.

Validation | All antibodies used in this study come from commercial manufacturers as specified in Supplementary Table 4 and below. Antibodies were used in accordance with manufacturer recommendations. All antibodies used were validated for appropriate species reactivity (i.e. mouse) and for use in flow cytometry applications/ Optimal concentrations were determined empirically. Specified dilutions, clones, and catalogue numbers are indicated. Antibody staining procedures are specified in the Method section of the paper.

Application Target Tag Clone Catalog # Notes Dilution Source
Mass Cytometry CD235a/b 141Pr HIR2 3141001B 1/100 Fluidigm
CD196 (CCR6) 141Pr G034E3 3141003A 1/100
CD19 142Nd HIB19 3142001B 1/100
CD62L 144Nd DREG56 304835/201144A Maxpar X8 Antibody Labeling Kit - 144Nd 0.5μg/stain Biolegend/Fluidigm
CD4 145Nd RPA-T4 3145001B 1/100 Fluidigm
CD8a 146Nd RPA-T8 3146001B 1/100
CD20 147Sm 2H7 3147001B 1/100
CD16 148Nd 3G8 3148004B 1/100
CD66a 149Sm CD66a-B1.1 3149008B 1/100
CD223 (LAG3) 150Nd 874501 3150016B 1/100
CD123/IL-3R 151Eu 6H6 3151001B 1/100
CD21 152 Sm BL13 3152010B 1/100
CD194 (CCR4) 153Eu 205410 3153013A 1/100
IgM 154Sm MHM88 314527/201154A Maxpar X8 Antibody Labeling Kit - 154Sm 1μg/stain Biolegend/Fluidigm
PD-I 155Gd EH12.2H7 3155009B 1/100 Fluidigm
CD38 156Gd HIT2 303535/201156A Maxpar X8 Antibody Labeling Kit - 156Gd 0.3μg/stain Biolegend/Fluidigm
CD11c 159Tb Bu15 3159001B 1/100 Fluidigm
CD14 160Gd M5E2 3160001B 1/100
CD183 (CXCR3) 163Dy G025H7 3163004B 1/100
IgD 164Dy IA6-2 348235/201164A Maxpar X8 Antibody Labeling Kit - 164Dy 0.2μg/stain Biolegend/Fluidigm
CD61 165Ho VI-PL2 3165010B 1/100 Fluidigm
CD27 167Er O323 3167002B 1/100
CD45RA 169Tm HI100 3169008B 1/100

CD3 170Er UCHT1 3170001B 1/100
CD185 (CXCR5) 171Yb RF8B2 3171014B 1/100
HLA-DR 174Yb L243 3174001B 1/100
CD274 (PDL1) 175Lu 29E.2A3 3175017B 1/100
CD56 176Yb NCAM16.2 3176008B 1/100
CD11b/Mac-1 209Bi ICRF44 3209003B 1/100
CD45 89Y HI30 3089003B 1/100
Ki67 172Yb B56 3172024B 1/100
FoxP3 162Dy PCH101 3162011A 1/100
Cisplatin 195Pt 201195 3µM/stain
Spectral Cytometry CD3 AF532 UCHT1 58-0038-42 1/60 Thermo Fisher Scientific
ICOS AF488 C398.4A 313514 1/100 Biolegend
ZEB2 AF700 FAB73782N-100UG 923328 1/60 Novus/R&D
CD39 APC Fire750 A1 328229 1/20 Biolegend
PD-1 BB515 EH12.1 565936 1/10 BD
OX40 BB700 ACT35 566560 1/10 BD
IgG BUV395 G18-145 564229 1/20 BD
CD19 BUV737 SJ25-C1 612757 1/60 BD
CD127 BUV750 HIL-7R-M21 747089 1/25 BD
CD62L BV480 DREG-56 566174 1/60 BD
Perforin BV510 dG9 308119 1/40 Biolegend
CD45RO BV570 UCHL1 304225 1/60 Biolegend
TIGIT BV605 A15153G 372711 1/30 Biolegend
CXCR3 BV650 G025H7 353729 1/40 Biolegend
CCR6 BV711 G034E3 353435 1/10 Biolegend
Helios Pacific Blue 22F6 137210 1/30 Biolegend
CD4 Pacific orange S3.5 MHCD0430 1/20 Thermo Fisher Scientific
FoxP3 PE PCH101 12-4776-41 1/20 Thermo Fisher Scientific
CX3CR1 PE Dazzle 594 2A9-1 341623 1/60 Biolegend
CD25 PE-Cy5 BC96 302607 1/20 Biolegend
Granzyme B PE-Cy5.5 GB11 GRB18 1/180 Thermo Fisher Scientific
LAG3 PE-Cy7 3DS223H 25-2239-41 1/10 Thermo Fisher Scientific
CD27 PerCP O323 302817 1/60 Biolegend
CD14 Qdot-705 TüK4 Q22137 1/300 Thermo Fisher Scientific
CD45 Qdot-800 HI30 Q10156 1/300 Thermo Fisher Scientific
CXCR5 Super Bright 436 MU5UBEE 62-9185-41 1/10 Thermo Fisher Scientific
Live/Dead Zombie NIR 423106 1/4000 Biolegend
Flow cytometry Nucleic acid stain YOYO-1 Iodide Y3601 0.5µM/stain Thermo Fisher Scientific/Invitrogen
Live/Dead Live/dead fixable Aqua Live/Dead Fixable Dead cell stain kit L34966 1/1000 Thermo Fisher Scientific/Invitrogen
CD3 Pacific blue UCHT1 48-0038-42 1/20 Thermo Fisher/eBiosciences
CD4 APC SK3 551980 1/10 BD
CD4 AF700 RPAT4 557871 1/100 BD
CD19 PerCP-Cy5.5 SJ25.C1 340951 1/20 BD
CD25 PerCP-Cy5.5 M-A251 560503 1/20 BD
41-BB PE-Cy7 4B4-1 309817 1/20 Biolegend
CD134 Biotin ACT-35 350024 1/20 Biolegend
IgD APC IA-62 348221 1/20 Biolegend
IgM PE-Cy7 MHM-88 314531 1/20 Biolegend
CD45RO FITC UCHL1 555492 1/5 BD
CD27 PE-Cy5 O323 15-0279-42 1/20 Thermo Fisher/eBiosciences
CXCR3 PE 1C6/CXCR3 550633 1/5 BD

Table S4: Antibodies for mass cytometry, spectral cytometry and flow cytometry analysis

Eukaryotic cell lines

Policy information about cell lines and Sex and Gender in Research

Cell line source(s)
The only cell line used in this study was the THP-1 cell line which was obtained from ADCC.

Authentication
The cell line was not authenticated.

Mycoplasma contamination
THP-1 cells were confirmed mycoplasma negative.

Commonly misidentified lines (See ICLAC register)
No commonly misidentified lines were used in this study.

Clinical data

Policy information about clinical studies

All manuscripts should comply with the ICMJE guidelines for publication of clinical research and a completed CONSORT checklist must be included with all submissions.

Clinical trial registration	Not applicable
Study protocol	the full study protocol is reported in the manuscript.
Data collection	Study subjects who presented with malaria were recruited and enrolled at Mfera Health Center in Chikwawa District under the auspices of the Malawi International Center of Excellence for Malaria Research. Recruitment and data collection (demographics, malaria infection status) were collected at the health center during monthly and interim sick visits over 18 months. Study recruitment started in June 2014 and the study was completed March 2017.
Outcomes	The number of clinical malaria episodes over 18 months was the primary outcome. Clinical malaria was defined as clinical symptoms consistent with malaria and a positive malaria blood smear (>2,500 parasites/µL), a standard definition used in malaria field studies. Secondary outcomes included asymptomatic malaria.

Flow Cytometry

Plots

Confirm that:

☐ The axis labels state the marker and fluorochrome used (e.g. CD4-FITC).

☒ The axis scales are clearly visible. Include numbers along axes only for bottom left plot of group (a 'group' is an analysis of identical markers).

☒ All plots are contour plots with outliers or pseudocolor plots.

☒ A numerical value for number of cells or percentage (with statistics) is provided.

Methodology

Sample preparation	All Sample Preparation: Frozen vials of PBMCs were placed in a 37°C water bath and gently agitated until ~90% was thawed. The vials were transferred to a tissue culture hood and 1mL of sterile warmed media (RPMI/20%FBS) was added to the PBMCs for 5 minutes before transferring drop-wise to a 10ml volume of warmed media (RPMI/20%FBS) containing 200U/ml of DNase I (Roche, Switzerland). After 10 minutes at room temperature, the PBMCs were centrifuged at 300g, at room temperature for 5 minutes. The pellets were washed twice with room temperature media (RPMI/20%FBS) before transferring to 96 well plates for staining with antibodies for CyTOF or spectral flow cytometry.<br><br>-For mass cytometry (CyTOF), PBMC samples were labeled with cisplatin, stained with heavy-metal conjugated Abs listed in Table S5, and barcoded following the manufacturer's protocol (Fluidigm, US) with minor modifications. Abs not commercially available as conjugated with specific heavy metal ions were conjugated using the ready-to-label antibody format and Maxpar Antibody Labeling kit (Fluidigm, US). Briefly, cells were stained with extracellular Abs in Maxpar staining buffer for 30 minutes on ice followed by addition of cisplatin (5µM in PBS) for 2 minutes at room temperature. Cells were washed twice in Maxpar staining buffer followed by fixation and permeabilization using the eBioscience FoxP3/transcription factor staining kit (Thermofisher Scientific) for intracellular staining and barcode labeling. Lastly, barcoded samples were combined for intercalation using Cytofix/Cytoperm buffer (BD) and 2% PFA/Ir solution for 30 minutes at room temperature. This was followed by a wash and resuspension of cells in MaxPar cell staining buffer with 125nM of Ir and storage at 4°C before running on the Helios CyTOF instrument.<br><br>-For spectral flow cytometry, participant's PBMCs were first stained with Live/dead Fixable Aqua (Thermofisher Scientific) for 30 minutes at room temperature. Cells were washed and incubated with human Fc block (BD) for 15 minutes on ice, followed by staining for extracellular markers with fluorochrome-conjugated antibodies (Table 5S) in FACS buffer (PBS, 1% FCS, 0.02% Sodium Azide, 2mM EDTA) and 50[illegible]l of BD Brilliant stain buffer (BD) for 30 minutes on ice. Cells were washed twice and then fixed and permeabilized with the eBioscience FoxP3/transcription factor staining kit (Thermofisher Scientific). Cells were resuspended in blocking permeabilization buffer (1% mouse serum, 100U/ml heparin, 0.2% BSA) for 20 minutes at room temperature before addition of fluorochrome-conjugated antibodies against intracellular markers (Table S5) on ice for 30 minutes. Cells were washed and resuspended in FACS buffer and stored at 4°C before acquisition on the 5 laser Cytek Aurora instrument (Cytek Biosciences).
Instrument	CyTOF Helios, FACSAria III and Cytek Aurora.
Software	Analysis was done using FlowJo v9 or v10 software (TreeStar).
Cell population abundance	Memory CD4+ T cells (CD45RO+)sorted for single cell analysis represented ~25% of PBMC. Purity was assessed to be >98% after sorting on a small sample. For ZEB2+ cells phenotypic analysis, these represented ~0.5-10% of the memory CD4+ T cells.

Gating strategy

In all plots, cell populations were from blood PBMCs, gated based on FSC-A and SSC-A and doublet exclusion. Further gating on lineage markers (i.e. CD3, CD4, CD8, CD11b, CD56) and transcription factors (FOXP3, was specified for each respective panel.

☒ Tick this box to confirm that a figure exemplifying the gating strategy is provided in the Supplementary Information.
